# Predicted Mercury Soil Concentrations from a Kriging Approach for Improved Human Health Risk Assessment

**DOI:** 10.3390/ijerph15071326

**Published:** 2018-06-25

**Authors:** David Imo, Holger Dressel, Katarzyna Byber, Christine Hitzke, Matthias Bopp, Marion Maggi, Stephan Bose-O’Reilly, Leonhard Held, Stefanie Muff

**Affiliations:** 1 Division of Occupational and Environmental Medicine, Epidemiology, Biostatistics and Prevention Institute, University of Zurich & University Hospital Zurich, Hirschengraben 84, 8001 Zurich, Switzerland; holger.dressel@usz.ch (H.D.); Katarzyna.Byber@usz.ch (K.B.); Christine.Hitzke@usz.ch (C.H.); Marion.Maggi-Beba@usz.ch (M.M.); 2 Division of Chronic Disease Epidemiology, Epidemiology, Biostatistics and Prevention Institute, University of Zurich, Hirschengraben 84, 8001 Zurich, Switzerland; matthias.bopp@uzh.ch; 3 Institute and Clinic for Occupational, Social and Environmental Medicine, University Hospital Munich, LMU Munich, WHO Collaborating Centre for Occupational Health, Ziemssenstraße 1, D-80336 Munich, Germany; Stephan.Boeseoreilly@med.uni-muenchen.de; 4 Department of Public Health, Health Services Research and Health Technology Assessment, UMIT (University for Health Sciences, Medical Informatics and Technology), Eduard-Wallnöfer-Zentrum 1, 6060 Hall i.T., Austria; 5 Department of Biostatistics, Epidemiology, Biostatistics and Prevention Institute, University of Zurich, Hirschengraben 84, 8001 Zurich, Switzerland; leonhard.held@uzh.ch (L.H.); stefanie.muff@uzh.ch (S.M.)

**Keywords:** human biomonitoring, geostatistics, kriging, children and mothers, health risk assessment, environmental epidemiology, mercury

## Abstract

Health-risks from contaminated soils are assessed all over the world. An aspect that many risk assessments share is the heterogeneity in the distribution of contaminants. In a preceding study, we assessed potential health-risks for mothers and children living on mercury-contaminated soils in Switzerland using human biomonitoring-values (HBM) and soil samples. We assessed 64 mothers and 107 children who had resided in a defined area for at least 3 months. HBM-concentrations for mercury in urine and hair were measured, a detailed questionnaire was administered for each individual, and more than 4000 individual mercury soil values were obtained in 2015. In this study, we aimed at investigating possible associations of mercury soil- and HBM-values by re-analyzing our data, using predictions of the mercury concentrations at the exact location of the participant’s homes with a kriging approach. Although kriging proved to be a useful method to predict mercury soil concentrations, we did not detect an association between mercury soil- and HBM-values, in agreement with earlier findings. Benefits of geostatistical methods seem to be limited in the context of our study. Conclusions made in our preceding study about potential health risks for the residential population are robust and not altered by the current study.

## 1. Introduction

Historically, mercury-contaminated sites are found all over the world, including in developed countries [1,2,3,4,5,6,7,8]. One example is located in and around the city of Visp in Switzerland, where mercury-containing wastewaters were led into a canal that discharges into the river Rhone. Often it is unclear, whether these contaminated sites pose a health threat or not. Performing a risk assessment for potential health risks from contaminated soils is ideally based on human biomonitoring (HBM) to assess the internal exposures [9] in combination with the assessment of external exposures by ambient monitoring of soil, air, water and/or food analyses [10]. 

In a preceding study [11], we assessed the risk from mercury-contaminated soils for children and mothers, living in an area with partly elevated mercury soil concentrations in the canton of Valais. Children are more likely to have contact with mercury in soils, as they tend to spend more time outside on the ground; thus, they are more likely to inhale mercuric vapors and play with soil and swallow it (hand- and object-to-mouth behavior). Mothers are the next most vulnerable group because of possible pregnancies and reproductive toxicity. 

The canton of Valais commissioned us with an expert opinion about potential health risks for the resident population. More than 4000 soil samples have been taken and analyzed for total mercury contents in the region of interest along the “Grossgrundkanal” canal between Visp and Raron in the canton of Valais in Switzerland. Nonetheless, out of 171 participants, we had missing soil values for 12 participants that were living on three different parcels, because the respective parcels have not been probed. On the other hand, for larger parcels, several values of probes across the area were available. In our prior study we used the value of the closest measurement point to the house where a participant was living as a proxy for the mercury concentration on the respective parcels (properties). We found no evidence that mercury soil levels pose a threat to human health for the studied population. In comparison with health-related guidance values and international reference values, the HBM results were unsuspicious. Furthermore, no evidence for an association between mercury soil and HBM values has been found [11].

However, the soil samples showed a high variability of mercury concentrations in their distribution within the parcels and within the concerned region, because mercury-containing sludge from the canal was dispersed spotty in the surrounding area. In general, high variability within soil samples of contaminated areas is a frequent problem [12,13]. 

The high fluctuations in mercury soil concentrations and the missing soil values for 12 participants were our driving rationale for further geostatistical analyses and predictions of soil concentrations. There are different geostatistical methods to appropriately deal with these variabilities and to predict local concentrations and distributions of pollutants [14,15,16,17]. One suitable method to cope with variability in soil samples is kriging [18]. Kriging is a geostatistical interpolation method used to smooth a surface and to predict specific soil concentrations. It is furthermore used frequently for different prediction models in epidemiological studies [19,20,21]. There are many studies, assessing health risks from mercury-contaminated soils using exposure modelling and/or geostatistical methods [22,23]. However, there are very few studies investigating exposures from contaminated soils using HBM and even fewer with individual soil samples [24,25,26]. To our knowledge, this is the first study combining HBM, individual soil samples and geostatistical methods in the context of environmental health.

In this study, we aimed at investigating possible associations of mercury soil and HBM values by re-analyzing the described data, using predictions of the Hg concentrations with a kriging approach [18]. Furthermore, our aim was to understand whether it is beneficial to perform further geostatistical analyses in the context of this environmental health study and if the overall conclusion of our recommendations given in our preceding study are robust.

## 2. Materials and Methods 

### 2.1. Study Population 

In a preceding study, we assessed the risk from mercury-contaminated soils for children and mothers, living in an area with partly elevated mercury soil concentrations [11]. For that study, we invited all mothers and their children, aged 2–11 years, living for at least 3 months in the residential areas for which soil samples were available, namely, in Visp Kleegärten, Visp West or Turtig. 171 participants were included in the study, of which 64 mothers and 107 children. The participation rate regarding all contacted and most likely eligible potential participants (*n* = 367) was 47%. The study was conducted in accordance with the Declaration of Helsinki, and the protocol was approved by the Ethics Committee of the Zurich canton and the canton of Valais (Project identification code: PB_2016-02410). Oral and written informed consent was obtained from all adult participants before they participated in the study. For all participants who are minors, oral and written informed consent was obtained from at least one of their parents before they participated in the study.

### 2.2. Collection of HBM-Data and Questionnaire Data

We took urine and hair samples and used a questionnaire to conduct interviews. The interviews were conducted at the cantonal Hospital of Valais in Visp between June and October 2015. The questionnaire included questions about demographics, occupation, education, socio-economic status, migration status, fish and vegetable consumption, the most recent time point when fish was consumed, number of amalgam fillings and other questions regarding potential mercury contact (e.g., broken energy lamps and thermometers). Urine and hair samples were analyzed for total mercury content at the laboratory of the Institute and Outpatient Clinic for Occupational, Social and Environmental Medicine of the University Hospital Munich. The laboratory is certified by G-EQUAS, the German external quality assessment scheme. The creatinine levels in urine were determined in the clinical routine system and mercury levels were adjusted accordingly. Samples that were outside of the World Health Organization (WHO) reference range for creatinine adjustment (0.3 to 3.0 g/L) [27] were excluded from the subsequent analyses. A detailed explanation of the procedure and collected data is given in our preceding study [11].

### 2.3. Study Site

**Figure 1:**
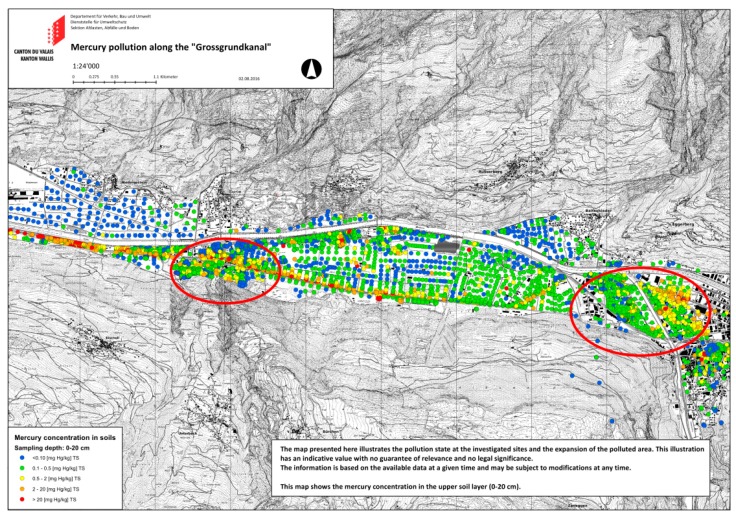
Mercury soil values (mg/kg) for the region of interest with a marked study area. Reproduced with permission from the canton of Valais, 2016. The left ellipse marks Turtig (Raron), the right ellipse marks Visp West and Visp Kleegärten. The Grossgrundkanal leads from Visp West to Turtig along the highest mercury soil values.

Figure 1 shows a map of the region of interest with mercury soil samples along the “Grossgrundkanal” canal between Visp and Raron. Each sample is indicated by a colored point. The highest values are found along the Grossgrundkanal. The inhabited parts of the region where participants were recruited from, namely, Visp West, Visp Kleegärten and Turtig, are indicated by a red circle.

### 2.4. Soil Data

Mercury soil values were obtained from the regional (cantonal) office of environmental protection. 4236 samples were taken (until the end of 2015) in this area and analyzed according to the Swiss regulations (VBBo). An engineering company performed the sampling. Samples were taken for the first 20 cm (0–20 cm) of soil, between 20 cm and 40 cm and in deeper layers. In our analyses, we included only the 3003 measurements of the first 20 cm because humans usually have the most contact with this layer, and data were available for nearly all the participants. The soil values and participants were linked via the respective parcel ID.

**Figure 2:**
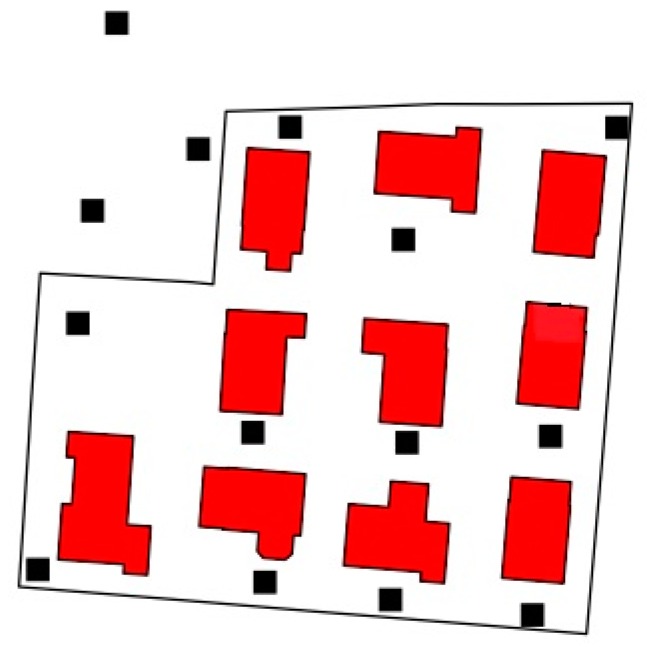
Sample points (black) on a large parcel with several houses (red).

For our new analyses we used the coordinates from the centroids of the houses the participants were living in to predict soil concentrations as appropriately as possible. In few cases, where centroid coordinates were not available from the official geoinformation, namely when several houses and/or several samples were located on one parcel (see Figure 2), we used the coordinates from the sample that was closest to the house, because this was then the most precise information that was available. The mercury values of the participants’ parcels ranged from 0.1 to 44.0 mg/kg. The mercury soil values in the region ranged from 0.1 to 690 mg/kg. Samples that were below the limit of determination (0.1 Hg mg/kg) were set to half of the respective limit of determination.

Both the mercury soil concentrations and the concentrations for mercury in urine had skewed distributions. Data were thus log transformed to accomplish an approximately Gaussian distribution. The original model is described thoroughly in the paper [11]. 

### 2.5. Distance to Canal

**Figure 3:**
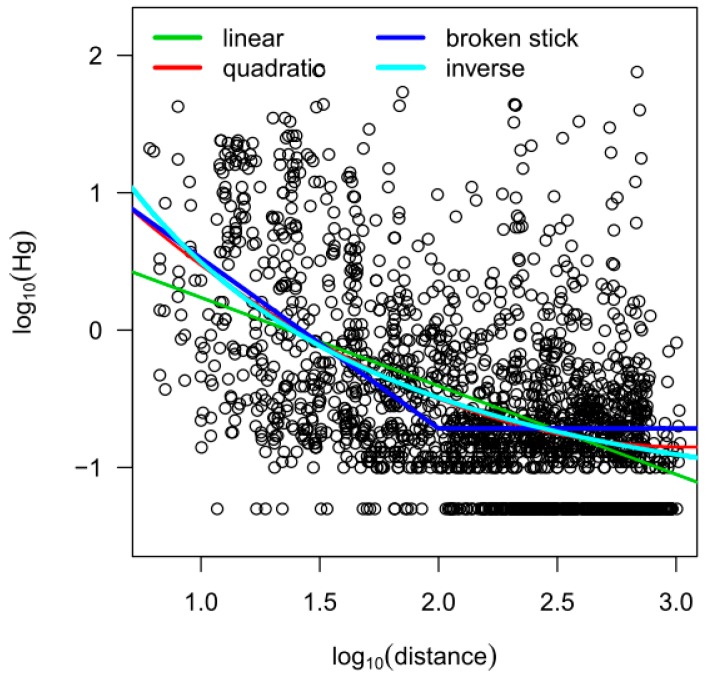
Mercury concentrations depending on the distance to the canal.

Historically, the canal was one major historic distributing factor of mercury-contaminated wastewaters. We therefore assumed that the distance to the canal had an influence on mercury soil concentrations, and generally, the highest concentrations are found close to the canal. We found the expected inverse relation, both graphically and with different regression models (see Figure 3 and Table 1). As the trend did not seem to be linear (green line), we added a quadratic term to the model (red line). Furthermore, we also used a “broken-stick” model (dark blue line) with breakpoint at log_10_ (distance) = 2, as well as an inverse model (1 + log of the distance (logdist))^−1^ (light blue line). Among these models, the quadratic model had the lowest Akaike information criterion (AIC) and Bayesian information criterion (BIC). Although the quadratic model has the disadvantage that the function again increases for large distances, which is practically implausible, we used this function to account for external drift in the geostatistical model described in the next subsection. 

### 2.6. External Drift Kriging

Kriging is a geostatistical method to smoothen the surface and to predict values at unsampled locations. It is frequently used in the context of soil contamination in order to assess environmental or human risks and for remediation purposes [15,28,29,30,31]. Given that the distance to the canal is correlated with the mercury soil concentrations, we used both a linear and a quadratic term as fixed effects in an external drift kriging model. 

The model we used for the data *Y*(*s_i_*) at location *s_i_* was as follows:

*Y*(*s_i_*) = *Z*(*s_i_*) + *ε* = *x*(*s_i_*)*^T^ β* + *B*(*s_i_*) + *ε_i_*,

where *Z*(*s_i_*) *= x*(*s_i_*)*^T^ β + B*(*s_i_*) is the signal, *x*(*s_i_*)*^T^ β* is the external drift with location-specific covariate vector *x*(*s_i_*)*^T^* and vector of regression coefficient *β*, and *B*(*s_i_*) is a stationary spatial Gaussian random field with zero mean and spatial covariance structure given by the stable exponential model and variance *σ*^2^ (the sill) that captures the auto-correlated variation. In addition, the model estimates the variance *τ*^2^ of the independent and identically distributed error term *ε_i_*, denoted as nugget effect, and the range parameter (*α*) of the variogram. A more detailed description of the model can be found in [32]. Given that the external drift kriging approach used here accounts for the distance to the canal, we assumed that the remaining spatial co-variance structure was isotropic, that is, the stationary spatial Gaussian random field depends only on the distance r between two measurement points. To this end, the covariance function *C*(*r*) = exp(−*r*^0.5^) from the stable exponential family was used. This choice was based on the rationale that the function might be continuous but not necessarily differentiable, because the contaminated material has been added in a pointwise manner. Note that the exponent of 0.5 was fixed here for computational efficiency and stability reasons, because when we estimated *γ* from model *C*(*r*) = exp(−*r^γ^*), all result were very similar and *γ* was very close to 0.5 (0.53), but computation became less stable and slow, in particular for the cross-validation runs described below. Finally, we also tested other covariance structures, such as the exponential (*C*(*r*) = exp(−*r*), a special case of the stable exponential model with *γ* = 1) and a Gneiting covariance [33], but semivariance and residual plots then clearly indicated model violations. For the prediction of model parameters we used the R-package georob [32], and fitted spatial linear models by robust REML [34]. 

**Figure 4:**
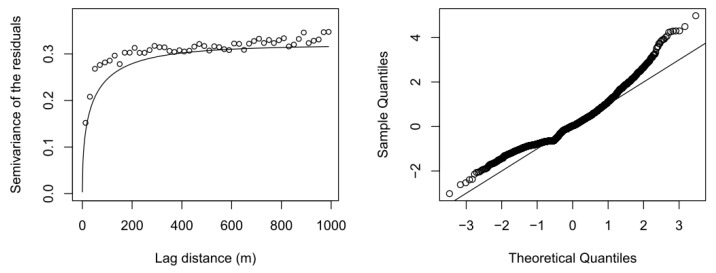
Variogram and QQ-plot for the external drift kriging model. (**a**) The variogram shows the relation between lag and semivariance in comparison to the method-of-moments estimator proposed by Matheron [35]; (**b**) The Q-Q-plot shows observed vs. theoretical quantiles [36].

The results for the predicted fixed effects and model parameters for the kriging model *σ*^2^ (variance), *τ*^2^ (nugget-effect) and range parameter *α* are given in Table 2. The variogram (Figure 4a) shows the relation between lag and semivariance in comparison to the method-of-moments estimator proposed by Matheron [35], and the Q-Q-plot shows observed vs. theoretical quantiles (Figure 4b) [36]. 

**Figure 5:**
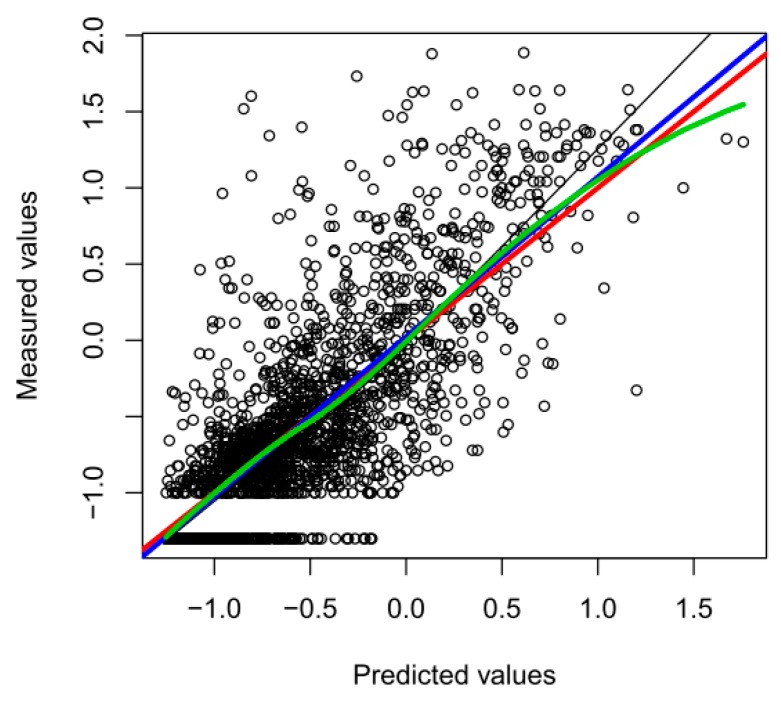
Correlation between measured and predicted soil concentrations. The green line indicates a function smoothened with locally-weighted smoothing LOESS [37], the blue line the least squares linear regression between measured and predicted soil values, and the red line represents the *y* = *x* axis.

To test the validity of our predicted soil concentrations, we did a 6-fold cross validation. In each iteration, we fitted the model on 5/6 of the data and compared the predicted values to the measured values for the remaining data points. The correlation between predicted and measured concentrations was as high as 0.77 (see Figure 5), although higher concentrations tended to be somewhat underestimated and low concentrations overestimated, as expected due to smoothing of the surface. In Figure 5, the green line indicates a function smoothened with LOESS [37], the blue line the least squares linear regression between measured and predicted soil values, and the red line represents the *y* = *x* axis. 

### 2.7. Kriging-Induced Measurement Error

Gryparis et al. [38] and Lopiano et al. [39] showed that smoothing techniques, such as kriging, introduce Berkson measurement error [40] into the variable that is predicted at locations other than where actual measurements are available. Consequently, a Berkson measurement error model accounts for error induced by the respective spatial misalignment. Muff et al. [41] showed that Integrated Nested Laplace Approximations (INLA) can be used for error modeling. For our analyses, we thus formulated a Berkson error model and fitted it with INLA. A key ingredient in formulating error models is the use of reasonable measurement error variances. The respective information was derived here from the standard errors that are given for the predicted values from the kriging approach. Note, however, that we ignored a possible spatial dependency of the error term.

### 2.8. Explanatory Variables

In the following, we describe which explanatory variables were included in our regression analyses of mercury levels in children and mothers. Predicted mercury (Hg) values in the soil (mg/kg) of the parcels on which the participants lived were log transformed to base 10 (Log_10_ Hg soil). The variable determination limit was used as an indicator if the respective soil value was below the determination limit of 0.1 mg/kg. Mother, as a binary indicator, was encoded as 1 if the participant was a mother and 0 otherwise. The indicator country of birth near the sea was 1 if the mother was born in Portugal, Croatia, Italy, France, Spain, or Turkey and 0 otherwise, reflecting that individuals from these countries tend to have different (usually higher) mercury biomonitoring values than individuals from Switzerland, presumably due to differences in dietary habits. The indicator variable eats vegetables from garden was 1 if vegetables from a garden in the region were consumed regularly and 0 otherwise, while smoking was a binary indicator for the smoking status of an individual. Sea fish represented the (square-root transformed) numbers of portions of sea food consumed in the last 30 days, including tuna (fresh or canned), salmon (smoked, filet, steak, fresh), sardines, shark, swordfish, cod, bass, flatfish (plaice, turbot, flounder, sole), haddock, and breaded fish products. Last time sea fish was used as an indicator if sea fish was consumed within the last 3 days, because this is the time interval, where a short-term increase in urinary mercury is expected. Amalgam fillings were counted and square root transformed (amalgam fillings). Hair dyeing within the last 6 months was used as indicator variable (hair dyeing). Finally, age (in years) at time when the questionnaire was filled in, was included. 

### 2.9. Statistical Analyses

In four separate regression analyses, the associations of mercury values in urine or hair with measured or predicted mercury soil concentrations (Log10 Hg soil) were evaluated. In each model, mothers and children were analyzed in a single model, but an indicator variable for mothers was added to account for potential differences between children and mothers. Furthermore, we accounted for the family effect by including family as an independent Gaussian random variable. In the models with creatinine-adjusted mercury in urine (µg/g creatinine) as the dependent variables, the main predictors were either Log10 Hg soil (measured) or Log10 Hg soil (predicted), with additional explanatory variables age, mother (indicator), number of amalgam fillings, number of sea fish meals, indicator for last time sea fish, smoking status, indicator for the determination limit, indicator for country of birth near the sea, and indicator if vegetables were eaten from the region. In the models with mercury in hair (µg/g) as the dependent variables, the main predictors were again either Log10 Hg soil (measured) or Log10 Hg soil (predicted). We adjusted for the same variables as for urine, but additionally included hair dyeing behavior, while the variable last time sea fish was excluded from these models because consuming sea fish within the last 3 days is not expected to directly affect mercury in hair, as it is a long-term measure. All analyses were conducted using R version 3.4.3 [42]. 

## 3. Results

**Figure 6:**
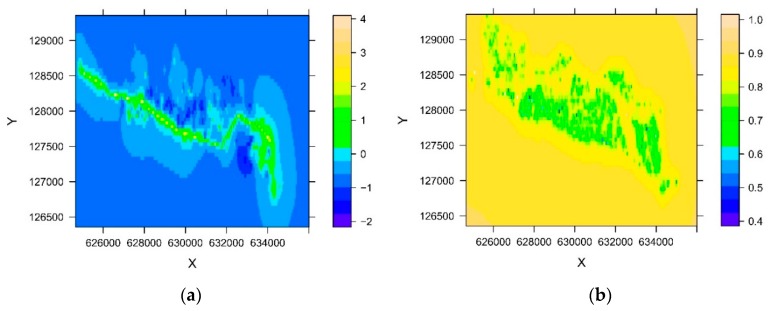
Predicted mercury soil concentrations in mg/kg (**a**) and standard error (**b**) for the concerned region.

Figure 6 shows the predicted mercury soil concentrations (Figure 6a) and the standard error (Figure 6b) along the Grossgrundkanal in the concerned region. Parcels that are further away from the canal have been sampled less. Hence, the standard error is higher for those parcels.

In our original analyses, we found no evidence for an association of mercury soil concentrations with urinary mercury concentrations (regression coefficient: 0.02; 95% CI: −0.06 to 0.10; *p* = 0.64) (see Table 3). In comparison to the analyses that included the predicted soil values from the kriging procedure, the results did not substantially differ (regression coefficient: 0.08; 95% CI: −0.23 to 0.42; *p* = 0.61) (see Table 4). 

Furthermore, we found no evidence for an association of mercury concentrations in soil with mercury concentrations in hair in our native model (regression coefficient: 0.05; 95% CI: −0.05 to 0.14; *p* = 0.32) (see Table 5). Using the predicted soil values instead of the measured values, did only marginally change the results (regression coefficient: 0.03; 95% CI: −0.11 to 0.17; *p* = 0.67) (see Table 6). 

To better communicate our results with a non-scientific audience, we categorized the results of our prior study in order of evidence for an association according to Martin Bland [43] (see Table 7). 

The categories of the results for the association with log-transformed mercury values in urine (µg/g creatinine) changed slightly. The *p*-values of “smoking” and “sea fish” increased from 0.005 to 0.02 and from 0.003 to 0.04, respectively. This implies a shift in category from “strong evidence” to “evidence”. The categories of the results for the association with log-transformed mercury values in hair (µg/g) did slightly change. The *p*-value of “country of birth near the sea” increased from 0.041 to 0.07. This implies a shift in category from “evidence” to “weak evidence”. Furthermore, “hair dyeing” and “mother (indicator)” shifted from the category “weak evidence” to “little or no evidence” (*p*-value: 0.072 to 0.15, and *p*-value: 0.095 to 0.15, respectively). 

## 4. Discussion

In this study, we found that the association between mercury soil concentrations and mercury concentrations in urine did not substantially change with the use of predicted soil concentrations from a kriging procedure. Furthermore, the association between mercury soil concentrations and mercury concentrations in hair did only change marginally. However, if an association was present, we would expect it mainly between mercury soil and mercury urine concentrations but less so with mercury hair concentrations as inorganic mercury is the predominant chemical form of mercury in the soils of the studied area. If humans take it up, the appropriate matrix to detect it is urine [44].

Although there are many sites with historical mercury contamination, there are only very few studies that assessed the external and internal concentrations to assess mercury-related health risks [24,25,26]. None of these studies found association between mercury soil and mercury HBM-values. To our knowledge, this is the first study comparing regression models with measured soil concentrations and predicted soil concentrations and HBM-values in the context of environmental health. 

The scenario where health risks from contaminated soils are assessed is quite common [45,46,47,48,49,50,51,52,53,54]. A frequent problem these scenarios share is the heterogeneity of the distribution of the contaminants in soils [11,12]. Universal kriging proved to be a useful method to predict reliable mercury soil concentrations, accounting for heterogeneity [55]. However, in practice the additional benefits of performing universal kriging for health risk assessments from mercury-contaminated soils seems to be limited in the context of our study. We are aware of the limitation that universal kriging smooths the highest values and outliers. Indicator kriging might be a suitable alternative that tackles this limitation [56]. 

The statements from our preceding study about possible health risks are robust and are not altered by these additional analyses. Nonetheless, when considering other chemicals that are more mobile, volatile, bioavailable and toxic, incorporating the prediction of soil concentrations may be beneficial for health risk assessments, especially, when less samples are available for the concerned area. However, we suggest that assessing the internal concentrations should be the first step in a risk assessment in such a scenario, and could be complemented and enhanced by additional modeling. 

A strength of the present study is the very high number of soil samples in combination with the high-quality geostatistical- and HBM-analyses. Our team of subject-matter experts was essential in gaining the high-quality results we obtained. The effect of well-known covariates (amalgam fillings, consumption of sea fish) is shown very clearly in the analyses [57,58]. 

The limitations of this study should also be mentioned. Although we included a Berkson error model in our analyses, using a mixed model of Berkson and classical error might have been a suitable option as well [59,60,61]. In addition, our error model did not account for potential spatial dependencies of the error [60,61].

To be consistent with our previous publication [11], and to be able to compare the results, we used log-transformed values of both the mercury soil concentrations and the concentrations for mercury in urine. However, the normal score transformation might often be more appropriate [62]. Nevertheless, our results were insensitive and thus robust upon this choice. 

Furthermore, the participation rate regarding all contacted and most likely eligible potential participants was 47%. This is in a normal range for such a study. However, with a higher total number of participants, it may have been possible to detect evidence for an association between mercury soil and mercury biomonitoring values. Nonetheless, we would expect the effect to be very low in comparison to the main drivers, such as amalgam fillings and fish consumption. 

## 5. Conclusions

To our knowledge, this is the first environmental health study comparing regression models with HBM-values as dependent variable comparing the influence of measured soil concentrations one the one hand, and predicted soil concentrations using a kriging approach on the other hand. This geostatistical approach using predicted soil concentrations may be applied in future risk assessments when the number of measurements is limited. The methods may be further refined, e.g., by using indicator kriging, or by taking into account spatial dependencies of the kriging-induced Berkson measurement error [39,61]. In our actual study setting, using predicted mercury soil concentrations and human biomonitoring we found no association between mercury soil- and human biomonitoring-values. 

## Figures and Tables

**Table 1:** Results of regression models that estimate the association between mercury concentrations and the distance to the canal, including the respective AIC and BIC values.

Model	Coefficient	95% CI	*p*-Value	AIC	BIC
Linear model					
logdist	−0.64	−0.69 to −0.59	<0.001	3435	3452
Quadratic model					
logdist (logdist)^2^	−1.91 0.31	−2.29 to −1.54 0.22 to 0.40	<0.001 <0.001	3393	3415
Broken stick model					
logdist, bp	−1.24	−1.33 to −1.15	<0.001	3423	3439
Inverse model					
logdist (1 + logdist)^−1^	0.16 6.90	−0.10 to 0.42 4.72 to 9.08	<0.001 <0.001	3399	3421

Abbreviations: CI: Confidence interval; AIC: Akaike information criterion; BIC: Bayesian information criterion.

**Table 2:** Fixed effects and model parameter of the kriging model.

Model Parameters	Estimates	95% CI	*p*-Value
Intercept	2.34	1.90 to 2.79	<0.001
log_10_ Hg	−2.23	−2.72 to −1.73	<0.001
log_10_ Hg^2^	0.38	0.24 to 0.51	<0.001
Sill *σ*^2^	0.32		
Nugget-effect *τ*^2^	0.003		
Range parameter *α*	46.67		

Abbreviations: CI: Confidence interval.

**Table 3:** Association with log-transformed mercury values (measured) in urine (µg/g creatinine).

Variable	Coefficient	95% CI	*p*-Value	Evidence for an Association
Amalgam fillings	0.33	0.24 to 0.42	<0.001	Very strong evidence
Last time sea fish	0.32	0.17 to 0.47	<0.001	
Age	−0.04	−0.06 to −0.02	<0.001	
Interaction age × mother	0.05	0.02, 0.08	<0.001	
Mother (indicator)	−0.97	−1.64 to −0.31	0.004	Strong evidence
Smoking	0.30	0.09, 0.50	0.005	
Sea fish	0.08	0.03, 0.13	0.003	
Log10 Hg soil	0.02	−0.06 to 0.10	0.64	Little or no evidence
Determination limit	−0.08	−0.25 to 0.09	0.37	
Country of birth near the sea	−0.01	−0.16 to 0.15	0.93	
Eats vegetables from region	0.07	−0.03 to 0.18	0.18	

Abbreviations: CI: Confidence interval.

**Table 4:** Association with log-transformed mercury values (predicted) in urine (µg/g creatinine).

Variable	Coefficient	95% CI	*p*-Value	Evidence for an Association
Amalgam fillings	0.28	0.20 to 0.35	<0.001	Very strong evidence
Last time sea fish	0.29	0.15 to 0.43	<0.001	
Age	−0.04	−0.06 to −0.02	<0.001	
Mother (indicator)	−0.74	−1.25 to −0.22	0.006	Strong evidence
Smoking	0.22	0.03 to 0.40	0.02	Evidence
Sea fish	0.06	0.01 to 0.11	0.04	
Log10 Hg soil (predicted) Country of birth near the sea Eats vegetables from region Family variance *σ*^2^_family_ Residual variance *σ*^2^*_ɛ_*	0.08 0.03 0.01 0.03 0.06	−0.23 to 0.42 −0.16 to 0.22 −0.11 to 0.13 0.01 to 0.06 0.04 to 0.07	0.61 0.78 0.81	Little or no evidence

Abbreviations: CI: Confidence interval.

**Table 5:** Association with log-transformed mercury values (measured) in hair (µg/g).

Variable	Coefficient	95% CI	*p*-Value	Evidence for an Association
Sea fish	0.17	0.11 to 0.22	<0.001	Very strong evidence
Country of birth near the sea	0.19	0.01 to 0.37	0.041	Evidence
Hair dyeing	−0.19	−0.39 to 0.02	0.072	Weak evidence
Mother (indicator)	−0.67	−1.46 to 0.12	0.095	
Log10 Hg soil (measured)	0.05	−0.05 to 0.14	0.32	Little or no evidence
Determination limit	−0.02	−0.22 to 0.17	0.81	
Eats vegetables from region	0.06	−0.06 to 0.18	0.31	
Smoking	0.12	−0.12 to 0.36	0.33	
Amalgam fillings	0.04	−0.06 to 0.14	0.43	
Age	0.01	−0.02 to 0.03	0.51	
Interaction age × mother	0.01	−0.02 to 0.04	0.46	

Abbreviations: CI: Confidence interval.

**Table 6:** Association with log-transformed mercury values (predicted) in hair (µg/g).

Variable	Coefficient	95% CI	*p*-Value	Evidence for an Association
Sea fish	0.15	0.10 to 0.21	<0.001	Very strong evidence
Country of birth near the sea	0.18	−0.01 to 0.37	0.070	Weak evidence
Hair dyeing	−0.15	−0.34 to 0.05	0.15	Little or no evidence
Mother (indicator)	−0.49	−1.13 to 0.16	0.15	
Log10 Hg soil (predicted)	0.03	−0.11 to 0.17	0.67	
Eats vegetables from region	0.06	−0.06 to 0.18	0.33	
Smoking	0.07	−0.16 to 0.30	0.54	
Amalgam fillings	0.03	−0.06 to 0.13	0.51	
Age	0.01	−0.02 to 0.03	0.33	
Family variance *σ*^2^_family_	0.01	0.01 to 0.04		
Residual variance *σ*^2^*_ɛ_*	0.11	0.08 to 0.16		

Abbreviations: CI: Confidence interval.

**Table 7:** Categories of *p*-values.

Evidence for an Association	*p*-Value
Very strong evidence	<0.001
Strong evidence	<0.01
Evidence	Between 0.01 and 0.05
Weak evidence	Between 0.05 and 0.1
Little or no evidence	>0.1
